# On the localization of reward effects in overlapping dual tasks

**DOI:** 10.1007/s00426-024-02054-4

**Published:** 2024-11-15

**Authors:** Leif E. Langsdorf, Daniel Darnstaedt, Torsten Schubert

**Affiliations:** Institute for Psychology, Martin Luther University Halle-Wittenberg, Halle, Germany

## Abstract

In dual-task (DT) situations, performance deteriorates compared with single-task situations. Such performance decrements are frequently explained with the serial scheduling of the response selection stages constituting a bottleneck. Proof of this assumption stems from the observation that response times for the second task (task 2; RT 2) increase with decreasing stimulus-onset asynchrony (SOA).

In this study, we investigated how the reward prospect for task 1 performance affects task 1 and task 2 processing. For that purpose, we relied on the psychological refractory period paradigm (PRP) as a chronometric tool, to determine the locus of the reward effect in the processing chain of both tasks.

We obtained improved task 1 and task 2 performance; as indicated by reduced RTs in the reward compared to the no reward condition of task 1 and task 2. Furthermore, the reward effect propagated at short SOA from task 1 onto task 2, suggesting that the locus of the reward effect can be pinpointed before or at the bottleneck of task 1. Importantly, the mean reward effect on task 1 was increased compared to task 2, thus indicating that parts of the reward effect were not propagated onto task 2, therefore affecting task 1 motor processes.

In Experiment 2, we tested for the locus of the effect propagation to task 2. Therefore, we implemented a difficulty manipulation of the response selection of task 2. The results indicate that the reward effect is propagated from task 1 onto the response selection stage of task 2.

## Introduction

Humans often execute two tasks at the same time or in close succession. In such dual-task (DT) situations, participants’ performance often deteriorates compared to when the same tasks are performed separately. The underlying cognitive architecture has long been investigated using DT paradigms such as the psychological refractory period (PRP) paradigm. In such PRP situations participants perform two temporally overlapping choice reaction time (RT) tasks, which are separated by a variable interval between them, the stimulus onset asynchrony (SOA). The situation usually results in decreased performance compared to single-task situations referred to as dual-task costs and to a performance pattern in which the response times on the second task (task 2) are increased the shorter the SOA between the tasks. The central bottleneck model explains these costs with the serial processing of the central response selection stages, while peripheral stages (i.e. perceptual and motor stages) are assumed to be processed in parallel. It is assumed that at short SOA the response selection and motor stage of task 2 wait until the response selection of the first task (task 1) has finished. This leads to an interruption of task 2 and explains why the reaction time to task 2 (RT2) is increased at short SOA and decreases the longer the SOA between tasks. The reaction time to task 1 (RT1) is assumed to be unaffected by the SOA variation.

Despite the general debate about the nature of the bottleneck being strategical or structural (Kieras & Meyer, 1997; Pashler, 1994), numerous factors and interventions have been identified that can modulate bottleneck processing, such as e.g. training (Ruthruff et al., 2006; Schubert & Strobach, 2018; Strobach et al., 2014), age (Hein & Schubert, 2004; Strobach et al., 2012), and different input and output modality combinations (Hazeltine et al., 2006; Stelzel et al., 2006). A further relevant question in this vein of research is whether and how reward prospect affects DT processing (Fischer et al., 2018; Han & Marois, 2013; Yildiz et al., 2013).

Previous work of our group investigated the location of reward effects in a PRP DT scenario, in which reward was selectively provided for task 2 performance, which according to DT theories represents the task that is interrupted due to bottleneck processing in task 1 (Langsdorf et al., 2022). In that study, participants perceived an instruction that they could earn a maximum potential reward of 72 Euro Cent per block if they performed fast and accurately on task 2 while maintaining a low error rate on task 1 performance. Interestingly, we observed performance benefits in the reward compared to the no reward condition already on task 1 processing, which indicates that participants’ reward prospects on task 2 improved the execution of the earlier to be processed non-rewarded task 1 and, only then, following improved the execution of task 2 (but see: Rieger et al., 2021).

Such an effect localization of reward-related task improvements to the processing stream of task 1 although the subsequent task 2 was rewarded has important implications. It indicates that the prospect of reward in PRP DT situations will not only affect the task associated with reward but that the reward-related task improvements can spillover to the non-rewarded task as well. The localization of potential reward effects in the processing chain of a DT situation can, thus, provide a clue for understanding the mechanisms of reward processing in DT situations; it can also contribute to the understanding of results from other studies investigating reward-related improvements in situations with multiple tasks and reporting inconclusive result patterns for rewarded and non-rewarded tasks.

For the current case, the results of Rieger et al. (2021) are particularly relevant. The authors reported that rewarding either mainly task 1 or task 2 performance (but with different size) in a PRP-like paradigm did not lead to reward effects on task 2 performance. In their analyses of the PRP task performance, the authors focused especially on those task conditions, in which participants had *not* responded to task 1 (no-go trials), but only to task 2 and did not find significant reward-related changes in the processing time of task 2. Considering previous findings of Langsdorf et al. (2022), it is conceivable that not responding to task 1 in the Rieger et al. (2021) study has impeded the emergence of reward effects on task 2 performance. This would be an explanation for the lacking reward effect on task 2 performance by Rieger et al. (2021), which is also at odd with the findings of other authors like Kleinsorge and Rinkenauer (2012). These authors reported the occurrence of reward effects in a non-rewarded task with a cued task switching situation in which a sudden short-termed presentation of a reward cue could have caused the allocation of processing resources to the non-rewarded task although reward boni were associated with the other task. Consequently, more research is required to foster evidence about the potential spillover of reward effects on non-rewarded tasks in multiple task situations and elucidate the mechanisms for their occurrence. In the current study, we aimed, therefore, to assess the localization of reward effects in a PRP DT scenario in more detail and focused especially on a situation in which reward was directly awarded to task 1 but not to task 2. This allowed us to assess the generalizability of our former findings of reward-effect localization in task 1 in a PRP DT situation by comparing the current findings with those of our former study (Langsdorf et al., 2022). This way we aimed to better understand the locus of reward effects in DT scenarios and, through this, to understand better the origin of potential spillover of reward effects between tasks in multiple task situations.

To outline the logic of the current study, we first discuss the previously obtained reward effects and explain the logic of the rationale, which led to the conclusion that the task 2 reward prospect affected task 1 processing and then spilled-over to the task 2 processing time (Langsdorf et al., 2022). That particular conclusion is based on *the effect propagation logic*, which predicts that a change in the processing time of pre-bottleneck and bottleneck processing in task 1 of a PRP situation will be propagated via the bottleneck mechanism onto task 2 RTs (Janczyk et al., 2019; Pashler & Johnston, 1989; Van Selst et al., 1999; Van Selst & Jolicoeur, 1997). Importantly, the response time effect on RT2 should be larger at short SOA than at long SOA because of the lacking bottleneck at the latter SOA; please, note that the lack of a bottleneck would prevent that the change of task 1 pre-bottleneck/bottleneck processing time propagates into task 2 time. In the former study, we obtained a main effect of reward on RT1 reflecting participants’ faster responses in the reward compared to no reward condition. In addition, we obtained shorter RT2 in the reward compared to the non-reward condition with larger reward effects onto RT2 at short compared to long SOA. Thus, these results of Langsdorf et al. (2022) are consistent with the assumption that participants’ prospect of reward on task 2 processing shortened the processing stages before or/at the bottleneck processing already in task 1 and that this effect propagated onto task 2 processing.

However, we obtained several additional findings (Experiment 1), which might suggest that the reward prospect on task 2 had not only affected task 1 pre-bottleneck and/or bottleneck processes in task 1 but also other processes; this, however, might suggest, that some portions of the reward prospect can bypass the bottleneck between tasks and affected task 2 processes *in addition* to the effects resulting from the propagation mechanism described before. For example, the obtained data pattern showed that the mean RT2 reward effect was significantly increased (*m* = 47 ms) compared to the size of the RT1 reward effect (*m* = 33 ms), which might indicate that there was an additional locus of the reward prospect directly on task 2 in addition to the reward locus on task 1 processing. Such a possibility is also supported by the observation that the task 2 reward effect at short SOA was significantly increased compared to the corresponding task 1 reward effect at short SOA (51 ms versus 28 ms). According to the effect propagation logic, the size of the reward effects on task 1 should be of the same magnitude, if the effects have propagated from task 1 to task 2 processing via the bottleneck. Therefore, the above-mentioned reward effect sizes are at odds with that prediction, and the larger reward effects at task 2 compared to that at task 1 indicate that (at least) part of the processing time reduction cannot be explained by a pure propagation account.

In addition, we obtained a significant RT2 reward effect at long SOA. Although numerically small (*m* = 21 ms) this effect cannot be explained by effect propagation from the reward-related task 1 processing time reduction because at long SOA there is no bottleneck interrupting the two tasks. Altogether, these findings could indeed indicate that at least part of the reward effects on task 2 did not result from an *in*direct effect propagation via the bottleneck but from a direct reward effect onto the task 2 processing chain, which, thus, has bypassed the bottleneck or affected directly the processing time in task 2. This would be indicative of a more complex pattern of reward prospects in overlapping DT tasks than an effect localization only on task 1 pre-bottleneck and bottleneck processes, which then spills over to the other processes of task 2.

Therefore, in the current study, we wanted to elucidate the location of potential reward effects in DT situations in more detail, by investigating the effects of a reward application on task 1 (instead of task 2); this allows us to assess the generalizability of reward effects resulting from a direct reward prospect to task 1 as compared to indirect reward effects resulting from the reward-assignment to task 2 as in our former study.

Additionally, we aimed to test which specific processes are affected in task 1 by the reward prospect during DT processing. In particular, we were interested in whether reward affects pre-bottleneck/bottleneck processing stages only, or whether post-bottleneck stages of task 1 can also be affected by reward. According to the effect propagation logic, propagation of reward effects on task 2 processing time can take place only in the first case thus causing spill over of reward effects but not in the second case.

Earlier findings on reward effects in single-task studies have provided evidence that reward can affect each of the processing stages along the processing chain in sensory-motor choice RT tasks, i.e. the perception, the response selection, or the motor stage of task 1. For example, in a study by Engelmann and Pessoa (2007), participants performed an exogenous spatial cueing task, in which participants reported the location of a peripherally cued target stimulus superimposed on either a face or a house stimulus. Before each trial a cue indicated the obtainable reward value. The results indicated a linear increase in detection sensitivity (d prime) as a function of incentive magnitude (Chiew & Braver, 2016; Engelmann, 2009; Engelmann & Pessoa, 2007; Kiss et al., 2009). Such findings are also underpinned by electrophysiological evidence, e.g., studies with event-related brain potentials with a high temporal resolution, which indicated increased early visual potentials for high-reward compared to low-reward targets (Nadig et al., 2019). While such effects would indicate that the application of reward affects early attentional and/or perceptual processes, other studies indicated that reward affects the response selection process. In particular, Chiew and Braver (2016) used an Erikson flanker task, in which cues indicated whether or not reward was obtainable, and if the upcoming task situation was congruent or incongruent. This resulted in an overadditive interaction of reward and task information improving task performance. Similar results were reported by other studies, suggesting a link between reward and effects on response selection (Etzel et al., 2016; Kennerley & Wallis, 2009).

Next to that, it is also conceivable, that the prospect of reward would affect motor processes during DT processing. In a study by Bundt et al. (2016), participants performed a horizontal Simon-task, in which a cue indicated whether a reward was obtainable or not. The authors showed that reward expectation led to enhanced motor preparation as indicated by reduced motor evoked potentials after cue presentation in cortical regions stimulated by transcranial magnetic impulses compared to no reward expectation. These and other findings indicate a close relation between motivation state and motor processing during task processing (Chiu et al., 2014; Hollerman et al., 1998; Schultz, 2000). Thus, it is conceivable that the application of reward affects motor processes instead or in addition to the perceptual and response selection processing in sensory-motor tasks.

**Fig. 1:**
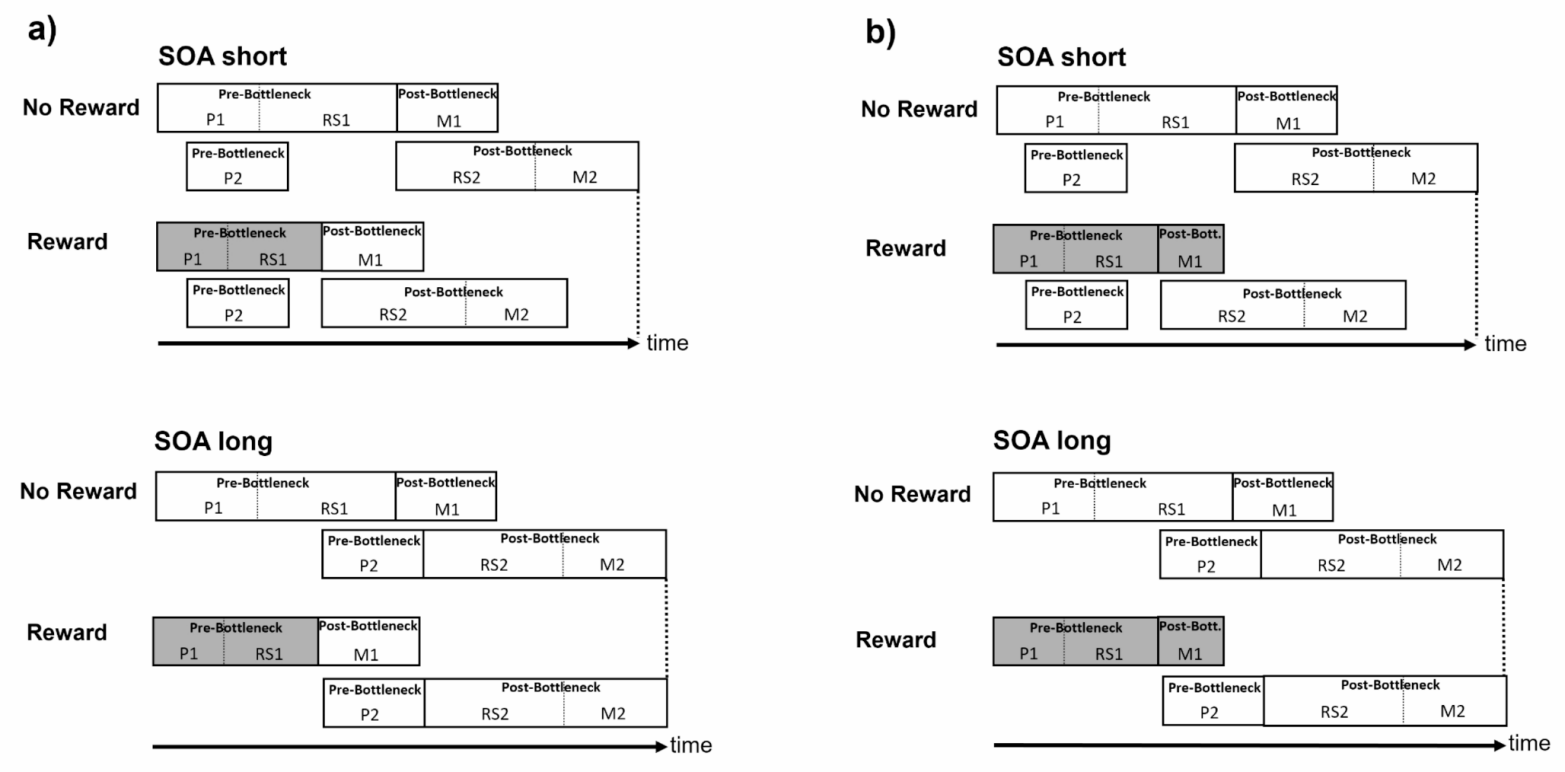
Reward improves processing of task 1. Panel (**a**) depicts the case when reward reduces the processing time of the pre-bottleneck and/ or bottleneck stages of task 1. The gray shaded areas of task 1 indicate that reward shortens the pre-bottleneck and/or bottleneck stages of task 1. This results in a reward effect on RT1 and an overadditive interaction of reward and SOA on RT2. Panel (**b**) depicts the case when reward reduces the processing time of the pre-bottleneck and/or bottleneck *and* the post-bottleneck stages of task 1. The gray shaded areas of task 1 indicate that reward shortens the pre-bottleneck and/or bottleneck stages and the post-bottleneck stages of task 1. This results in an overadditive interaction of reward and SOA on RT2. And increased reward effects on RT1 compared to RT2. Pre-Bottleneck stage of task 1 comprises: P1 = perception stage of task 1; Bottleneck stage of task 1 comprises: RS1 = response selection of task 1. Post-Bottleneck stage of task 1 comprises M1 = Motor stage of task 1; Pre-Bottleneck stage of task 2 comprises: P2 = perception stage of task 2; Bottleneck stage of task 2 comprises: RS2 = response selection stage of task 2; Post-Bottleneck stage of task 2 comprises: M2 = motor stage of task 2

In the present study, we applied the effect propagation logic in combination with a direct reward prospect on task 1 performance to assess (a) the reward effect on the DT performance on task 1 and task 2, and (b) to localize in more detail the specific processing stages of task 1, which are affected by a direct reward prospect on task 1. As can be seen in Fig. 1 different hypotheses can be distinguished in such an investigation.

For example, according to the findings of our former study (Langsdorf et al., 2022), it is conceivable that a direct reward prospect for task 1 shortens the processing time of the pre-and/or bottleneck processing stages of task 1. The locus of these potential reward effects would correspond to the outlined effects of reward on the perception and/or the response selection processes. Importantly, if that was the case, we would expect effect propagation of the reward effect at short SOA onto task 2, resulting in a reward effect at short but not at long SOA. This should be reflected by an overadditive interaction of SOA and reward on RT2 as can be seen in Fig. 1a. Importantly, the size of the reward effects on task 1 and task 2 should be identical if the reward prospect exclusively leads to a shortening of the processing duration of the pre-bottleneck and bottleneck processing time in task (1) In that case, each ms of processing time reduction in task 1 should be propagated onto the processing time in task (2) According to Pashler and O’Brien (1993), such a prediction would also be consistent with the finding of an increased interdependency of RT2 on RT1 at short SOA. In contrast, the RT2 interdependency on RT1 should decrease with increasing SOA as no bottleneck emerges between both tasks. Therefore an analysis of the relationship between RT1 and RT2 can provide additional evidence for the effect propagation between tasks (for details see the results section).

However, as an alternative a direct reward prospect on task 1 could lead to an effect localization on post-bottleneck stages in task 1, i.e. the motor stages of task 1. Indeed such an effect pattern would be discrepant with the earlier findings of Langsdorf et al. (2022), in which task 2 was rewarded and the reward effect on task 1 resulted rather from an indirect reward effect on task 1. However, the fact that in the current experiment, the reward prospect is directly allocated to task 1 can cause a more efficient reward localization onto task processing including the post-bottleneck stages in task (1) In the case of a reward effect location at the post-bottleneck stages of task 1, we would expect that the amount of the reward effect on task 1 is not the same as the one on task (2) As an extreme situation, consider the case that the whole reward effect on task 1 is allocated to the post-bottleneck stage of task 1. If that was the case, then no reward-related reduction of task 1 processing time would be propagated onto task 2 processing via the bottleneck because the reward effect emerges after the bottleneck. As a result, we would expect only a reduction of RT1 in the reward compared to the no reward condition but no reward effect on RT2.

Important to note that a further situation is conceivable, in which reward shortens pre-bottleneck/bottleneck *and*,* in addition*, the post-bottleneck stages of task (1) As can be seen in Fig. 1b, this particular situation would be characterized by a combination of both effect patterns: namely by larger reward effects at short compared to long SOA on RT2 resulting from effect propagation of the reward-related reduction in task 1 chain to task 2 and, *in addition*, by larger reward effects on task 1 compared to task (2) The latter (i.e. larger task 1 than task 2 effects) would emerge if the task 1-related reward effect would not completely be propagated into the task 2 RT because part of the reward-related shortening of the task 1 processing time would be located at post-bottleneck stages; that part of the processing time reduction would not be carried over to task 2 via the bottleneck as can be seen in Fig. 1b.

In addition to the location of reward effects in task 1, we also investigated which specific processing stages of task 2 are processed earlier as a result of the reward allocation onto task 1. This question is open because different bottleneck accounts (i.e., Meyer & Kieras, 1997; Pashler, 1994) would predict that the task 2 processing chain is interrupted at different processing stages and therefore, a potential effect propagation from task 1 onto task 2 would operate via different target processes in the task 2 processing chain. In more detail, according to accounts assuming a central bottleneck (Pashler, 1994), the effect propagation from task 1 to task 2 should cause an earlier start of the response selection stage in task 2, while accounts assuming a peripheral bottleneck (Meyer & Kieras, 1997) would assume that the effect propagation should cause an earlier start of the motor stage in task 2, while the start of the response selection processes in task 2 would not be affected. We will come back to this issue and the related hypotheses when introducing Experiment 2.

## Experiment 1

In Experiment 1, we tested to which degree the selective application of a reward prospect to task 1 affects participants’ task 1 and/or task 2 performance. Furthermore, we investigated which processing stages of task 1 are affected by a direct reward application to task 1 performance.

To this end, we administered an auditory-visual DT with three SOAs and instructed participants that they could earn a monetary reward if their response to task 1 was fast and accurate while maintaining low error rates in task 2. Note that participants received their performance-contingent reward at the end of the experiment; therefore, it is the expectation of a potential reward and not the actual reception in a given trial that would affect participants’ performance. For the sake of brevity, we will refer to this expectancy of a reward simply as the reward effect.

According to the assumption that the reward prospect for task 1 would exclusively affect the pre-and/or bottleneck processing stages of task 1, we should expect a reduction of RT1 and an effect propagation onto RT2. In detail, the effect propagation would result in a reward-related reduction of RT2 at a short SOA, which would be of the same size as that on task1, and no reward-related reduction of RT2 at a large SOA, where no task 2 interruption is taking place.

A reward effect on task 1 post-bottleneck processing, i.e. the motor stage of task 1 should lead to a reduction of the RT1, which would not propagate onto RT2. This in turn would be reflected by a larger size of the reward-related reduction of RT1 compared to RT2.

Worthwhile to note that reward can affect both, namely pre-and/or bottleneck processing stages and post-bottleneck stages of task (1) In that case, we would expect an overadditive reward X SOA interaction on RT2 suggesting reward effect propagation from task 1 to task 2; in addition (but opposite to the case of an exclusive task 1 pre-bottleneck effect), we should find larger reward effects onto RT1 compared to the reward effects on RT2 even at short SOA, where effect propagation takes place but not all of the reward effect in task 1 can be transmissed to task (2) This is so because part of the reward effect in task 1 would emerge after the bottleneck and would not be transmissed to task 2.

### Materials and methods

#### Participants

Twenty-four healthy participants (19 *female*; *mean* (m) age = 24.35 years) were invited to take part in the experiment after obtaining written informed consent and were debriefed after the session. We chose this particular sample size based on a priori power analyses obtained with the G*Power program (Faul et al., 2007). We conducted a power analysis for the interaction effect of reward (no reward and reward) and SOA (100 ms, 300 ms, or 900 ms) on RT2. Conceptualizing, the interaction as the main effect of SOA on the differences between no reward and reward conditions. For G*Power we defined the parameters as follows: Test family: F test; statistical test: ANOVA: Repeated measures, within factors; Type of power analysis: a priori; Effect size *f*: 0.67 (which corresponds to an effect size of *η*_*p*_*²* = 0.31, based on Langsdorf et al., 2022); α error prop: 0.001; Power (1-β error prob): 0.99; Numbers of groups 3; Number of measurements: 2; Corr. Among rep measure: 0.5; Nonsphericity correction ε: 1. The calculated sample size amounts to *N* = 24 for Experiment 1^1^. The experimental protocol conformed to the declaration of Helsinki. All participants were right-handed, German native speakers, and had normal or corrected to normal vision. Furthermore, Participants could choose between 4 Euro or course credit as a general payment, which was added by the performance-dependent amount of monetary reward (see below).

#### Apparatus and stimuli

Participants performed a PRP dual task consisting of an auditory and a visual choice RT task. Stimuli for the auditory task comprised of three sine-wave tones with a frequency of 250, 500, or 1000 Hz presented for 200 ms via headphones. Participants responded to the low-, middle-, and high-pitched tones by pressing the ‘Y’, ‘X’, and ‘C’ keys of a QWERTZ keyboard with the ring, middle, and index fingers of their left hand, respectively. For the visual task, one of three digits (*1*, *5*, or *9*) was presented centrally on a computer screen with a visual angle of 52° x 0.31° at a viewing distance of 80 cm. Visual stimuli appeared for 200 ms and participants responded to the digits in ascending order by pressing the keys ‘M’, ‘,’, and ‘.’ of a QWERTZ keyboard with the index, middle, and ring finger of their right hand. Participants were instructed to first respond to the auditory and then to the visual task. Every trial started with the presentation of a fixation cross at the center of the screen for 1000 ms followed by a blank interval for 500 ms. Subsequently, the auditory stimulus was presented for 200 ms, followed by the visual stimulus for 200 ms, separated by an SOA of either 100 ms, 300 ms, or 900 ms. After a response to both target stimuli or a maximal response duration of 3000 ms, an intertrial interval of 500 ms followed before the start of the next trial. Participants received the feedback “Falsch” (german for wrong) for 500 ms if either one or two of their responses were erroneous. If their response to either target exceeded the maximal response duration, the feedback “Zu langsam” (german for too slow) was presented for 500 ms.

#### Design and procedure

We applied a two-factor within-subjects design with reward and SOA as independent variables. Each block consisted of 27 trials resulting from the combination of 3 SOAs (100 ms, 300 ms, 900 ms), 3 auditory stimuli (250 Hz, 500 Hz, 1000 Hz), and 3 visual stimuli (1, 5, 9). In total 12 DT blocks were presented, and the blocked application of reward resulted in 6 reward and 6 no reward blocks. In sum, this resulted in 324 experimental trials. The procedure was as follows: The experiment started with a single-task practice phase in which participants performed 12 single-task trials for each component task (auditory and visual). The timing of these single-task trials was similar to DT trials with the exception that only one target stimulus was presented and only one response was required. These single-task trials were followed by two blocks of 27 trials of DT practice. At the start of the DT practice, Participants were instructed to respond to task 1 as soon as it was presented (Ulrich & Miller, 2008). Subsequently, the experimenter verbally instructed the participants using a standardized instruction that their task 1 performance was rewarded. And that they could earn 72 Euro Cent per block if their response to task 1 was fast and accurate, while their task 2 performance was not rewarded (however to mind low error rates for task 2). The information on whether or not a reward was obtainable was again presented before each block. In particular, to obtain a monetary reward of 72 Euro Cent per block, the RT1 as well as the error rates for task 1 have to be fast and accurate while considering low error rates for task 2. Participants’ thresholds for earning a reward were calculated based on their mean RT1 performance and their mean error rates in reward blocks, both indices in a given reward block were compared to these thresholds, to decide whether or not participants receive a reward. For the first reward block, we set a pre-defined deadline of 850 ms for task 1 performance as well as 89% accuracy, based on previous studies (Langsdorf et al., 2022; and pilot studies). If either participants’ mean RT1 or their mean error rates met the pre-defined threshold, they would receive 72 Euro Cent. If none of their performance measures were below the criterion measures, they received no reward. Thereafter, the reference RT1 was updated by averaging the pre-defined deadline (850 ms) and the mean RT1 of the previous reward block. Similarly, the mean error rate was updated. After each block, participants received feedback about their mean RT1 and percentage of correct trials, and for reward blocks, whether they earned a reward (and how much reward they had earned so far). The order of the 6 reward and 6 no reward blocks was randomized. Importantly, participants were naïve about the threshold computations for obtaining a reward.

**Statistical Analysis** Mean RTs and error rates were analyzed separately for RT1 and RT2 using an ANOVA with the within-subjects factors reward and SOA. A significance threshold of 5% was used for all analyses. The *p* values of the ANOVAs were adjusted according to the Greenhouse-Geisser correction when necessary. For the RT analyses, trials with at least one erroneous response (*m* = 7%) and outliers that deviated more than +/- 2.5 SD for each participant and factor combination (*m =* 2%) were excluded from the data set. Furthermore, trials were excluded that met the criterion of response grouping (RT2 – RT1 + SOA) < 200 (Miller & Ulrich, 2008). The data set of one participant had to be excluded due to technical issues, resulting in 23 data sets for further analysis.

### Results

#### Task 1

**Fig. 2:**
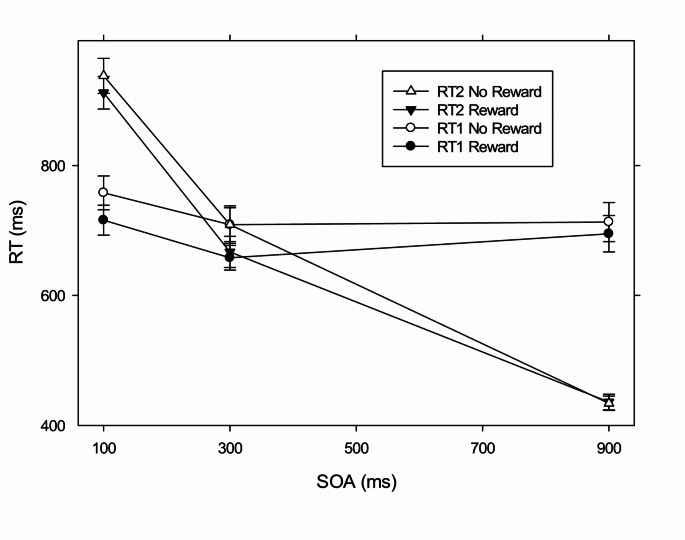
Mean RT1 and RT2 as a function of SOA and reward for Experiment 1. Error bars represent the standard error of the mean

We first tested for the effects of reward on task 1 performance. We obtained a significant main effect of the factor reward, *F*(1, 22) = 24.762, *p* < .001, η_p_² = 0.530. Participants’ RT1 was reduced in the reward (*m* = 690 ms) compared to the no reward (*m =* 726 ms; see Fig. 2) condition. Furthermore, we obtained a significant main effect of the factor SOA, *F*(1.236, 46) = 5.203, *p* < .024, η_p_² = 0.191, on RT1. Such effects of SOA on task 1 are often explained by participants’ tendency for response grouping (Strobach et al., 2015a; Ulrich & Miller, 2008). The interaction of the factors Reward × SOA was marginally significant, *F*(2, 44) = 3.079, *p* = .056, η_p_^2^ = 0.123. Further tests revealed a trend toward a larger reward effect at SOA 100 (*m* = 41 ms) compared to SOA 900 (*m* = 19 ms), t(22) = 1.541, p =. 066; and a larger reward effect at SOA 300 (*m* = 51) compared to SOA 900, t(22) = 2.526, *p* = .009. While the reward effects at SOA 100 and SOA 300 were not different, t(22) = − 0.779, *p* = .222.

##### Mean RT1/RT2 as a function of reward and SOA

For the error rates in task 1, we observed a significant main effect of the factor reward, *F*(1, 22) = 10.666, *p* < .004, η_p_^2^ = 0.327. The error rates were lower in the reward (*m* = 7%) compared to the no reward (*m =* 11%) condition. Furthermore, we observed a significant main effect of the factor SOA, *F*(1, 22) = 18.216, *p <* .001, η_p_^2^ = 0.453. The error rates were higher for SOA 100 (*m* = 13%) compared to SOA 300 (*m* = 7%) and SOA 900 (*m* = 5%). The interaction of the factors Reward × SOA, *F*(1.713, 44) = 1.713, *p* = .201, η_p_² = 0.072 did not reach significane.

#### Task 2

We observed a significant main effect of the factor reward, *F*(1, 22) = 12.876, *p* < .002, η_p_^2^ = 0.369 on RT2. Participants responded faster in the reward (*m* = 672 ms) compared to the no reward (*m* = 692 ms; see Fig. 2) condition. Furthermore, we found a significant main effect of the factor SOA, *F*(1.364, 44) = 442.265, *p* < .001, η_p_^2^ = 0.953. RT2 increased from SOA 900 (*m* = 435 ms) to SOA 100 (*m* = 925 ms), indicating the typical PRP effect (Pashler, 1994; Schubert, 1999).

Importantly, we observed a significant overadditive interaction of the factors reward and SOA, *F*(2, 44) = 5.496, *p* < .007, η_p_^2^ = 0.200 on RT2. Pairwise comparisons showed a significantly larger reward effect at SOA 100 (*m =* 27 ms) compared with SOA 900 (*m* = -2 ms), *t*(22) = 2.429, *p* < .024. While the reward effect at SOA 100 (*m* = 27 ms) was not different compared to the reward effect at SOA 300 (*m* = 38 ms), t(22)=-0.924, *p* = .183. This overadditive interaction of reward and SOA on RT2 indicates that the reward effect propagates from task 1 onto task 2. This is in line with previous evidence (Langsdorf et al., 2022), as well as with the assumptions described in the introduction part, and demonstrates that the reward application to task 1 affects the pre-and/or bottleneck processing stages of task 1. Further analysis will focus on potential additional effects of reward on other task 1 processing stages (see below).

For the error rates in task 2, the factor reward reached significance, *F*(1, 22) = 9.49, *p* < .005, η_p_^2^ = 0.301. The error rates in the reward (*m* = 5%) compared to the no reward (*m =* 8%) condition were reduced. The effect of the factor SOA reached significance, *F*(1, 22) = 3.609, *p <* .035, η_p_^2^ = 0.141. This indicated increased errors during SOA 100 (*m* = 8%) compared to SOA 300 (*m* = 5%), but not compared to SOA 900 (*m* = 7%). The interaction of the factors Reward × SOA, *F*(2, 44) = 1.879, *p* = .165, η_p_² = 0.079, did not reach significance.

**Table 1:** Mean rates of errors for task 1 and task 2 in % (and standard deviation) from experiment 1 as a function of reward and SOA

				Experiment 1			
		Error rates					
		reward			no reward		
SOA		Task 1	Task 2		Task 1		Task 2
100		9.98% (6.06%)	6.12% (5.87%)		16.18% (15.75%)		10.63% (7.80%)
300		5.80% (5.78%)	5.15% (6.19%)		8.86% (8.11%)		5.31% (7.80%)
900		4.19% (4.07%)	4.99% (4.27%)		6.28% (5.85%)		8.70% (6.94%)

### Relationship between RT1 and RT2

The subsequent analysis focused on the relationship between RT1 and RT2 in order to investigate in more detail the effect propagation between task 1 and task 2. The assumption that effect propagation had taken part from task 1 to task 2 processing time predicts a robust interdependency of RT2 on RT1 at shorter SOAs as a slower response to task 1 should lead to a slower response to task 2 due to the bottleneck mechanism. In contrast, at longer SOAs this interdependency should decrease as with reduced temporal overlap no bottleneck emerges between the tasks. To investigate the relationship between the speed of both responses we relied on the approach of Pashler and O’Brien (1993). For that purpose, RT1 was rank-ordered and split into quintiles for each factor combination. Subsequently, the mean RT2 of the corresponding factor combination was computed. For all points mapped on the plot, the value on the y-axis denotes the mean RT2 for those trials for which the RT1 lies within a particular quintile, while the value on the x-axis identifies the mean RT1 for this particular quintile.

**Fig. 3:**
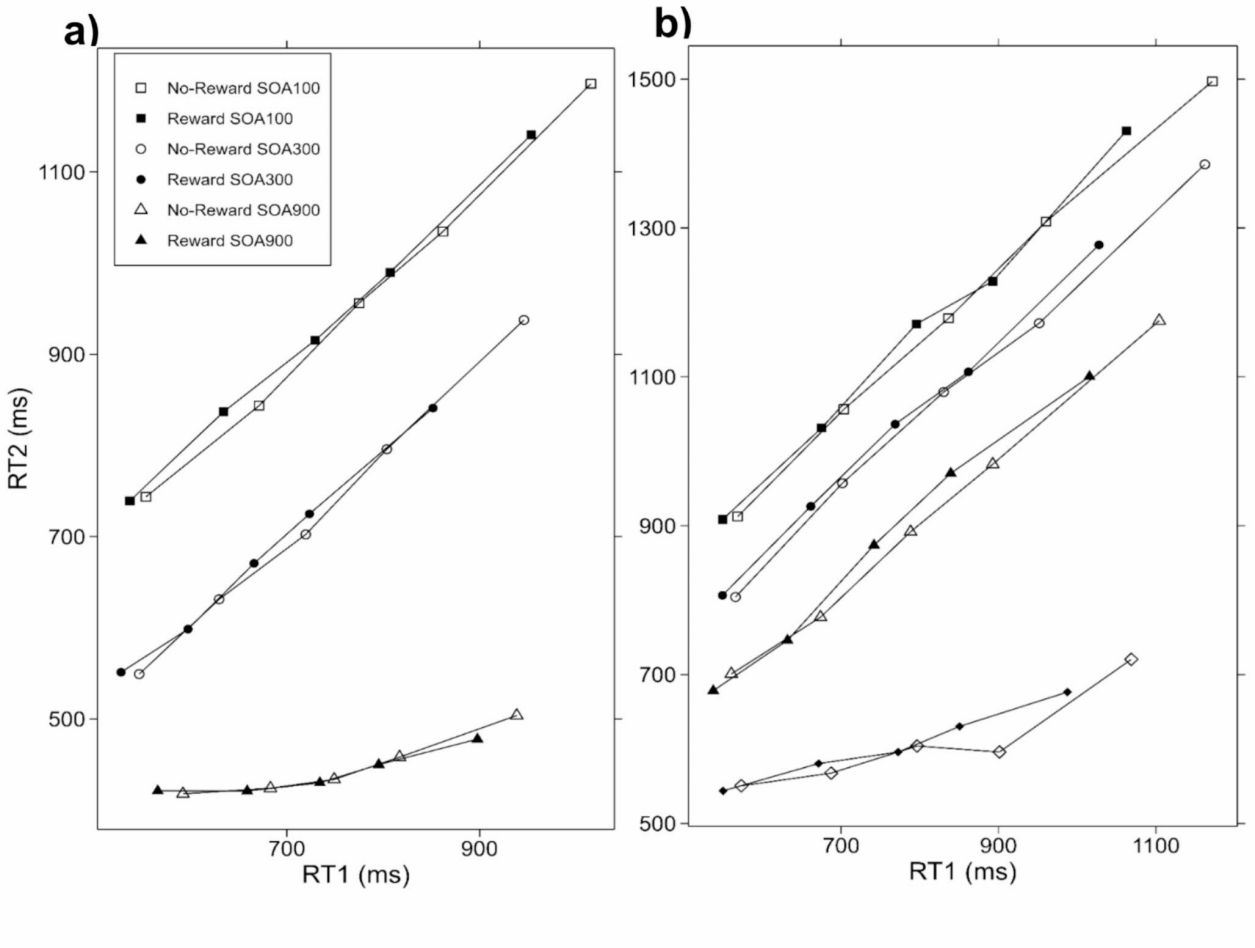
Mean RT2 as a function of reward, SOA, and RT1 (broken into quintiles). Panel **a**) represents the data from Experiment (1) Panel **b**) represents the data from Experiment (2) The legend for panel b: squares denote SOA 50, circles denote SOA 150, triangles denote SOA 300, and diamonds denote SOA 900. Filled symbols represent the reward condition and empty symbols represent the no reward condition

Figure 3a shows that an increase in RT1 led to a rise in RT2 as well. Most importantly, as SOA was reduced from 900 ms to 100 ms, the dependency of RT2 on RT1 increased. This observation was verified by the results of an ANOVA with RT2 as the dependent variable and the factors reward, SOA, and RT1 broken into quintiles; here, we obtained a significant interaction of the factors SOA and quintile, *F*(8, 176) = 67.61, *p* < .001, η^2^ = 0.137.

Moreover, as depicted in Fig. 3a we obtained a significant interaction of the factors reward and SOA, *F*(2, 44) = 3.43, *p* < .041, η^2^ = 0.004, suggesting larger reward effects at shorter compared to longer SOAs on RT2. This result replicated the finding obtained from our previous analysis of task 2 performance.

In addition, we found several other effects. Figure 3a displays that the reward effect was increased for larger RTs compared to smaller RTs, which is reflected by a significant interaction of the factors, reward, and quintile, *F*(4, 88) = 7.96, *p* < .001, η^2^ = 0.006; this is consistent with a proposal of Hübner and Schlösser (2010) that the prospect of reward can reduce lapses of attention of participants, which are usually among the longer RTs.

We also obtained a significant main effect of the factor SOA, *F*(2, 44) = 381.85, *p* < .001, η^2^ = 0.708 and a main effect of the factor quintile, *F*(4, 88) = 131.62, *p* < .001, η^2^ = 0.355. The factor reward reached significance, *F*(1, 22) = 12.94, *p* < .002, η^2^ = 0.011, indicating shorter RTs in the reward compared to the non-reward condition.

### Comparison of reward effects on task 1 and task 2 performance

To test whether the reward prospect on task 1 affected the post-bottleneck stages of the task 1 processing chain in addition to the effects on the pre-bottleneck and bottleneck stages, we compared the size of the reward-related RT reductions in task 1 and task 2. For that purpose, we calculated an additional ANOVA across the RTs in the two tasks with the factors task (task 1 vs. task 2), SOA, and reward. The factor task did not reach significance, *F*(1, 22) = 2.215, *p* = .151, η_p_² = 0.091, indicating no differences in processing speed between task 1 (*m* = 708 ms) and task 2 (*m* = 682 ms) RTs. The factor reward reached significance, *F*(1, 22) = 20.235, *p* < .001, η_p_² = 0.479, reflecting shorter RTs in the reward (*m =* 682 ms) compared to the no-reward condition (*m* = 708 ms). We further obtained a significant main effect of the factor SOA, *F*(1.460, 32.125) = 181.438, *p* < .001, η_p_² = 0.892.

Most decisively for the question of a reward-effect on the post-bottleneck stage in task 1, we obtained a significant interaction of the factors reward and task, *F*(1, 22) = 19.683, *p* < .001, η_p_² = 0.472, which reflects the fact that the reward prospect led to a larger reduction of the RTs for task 1 than for task 2, m = 37 ms versus m = 21 ms, t(22) = 3.049, *p* < .006, for task 1 and task 2, respectively. Separate analyses at the separate SOAs indicated that reward prospect led to a larger reduction of RT1 compared to RT2 at each separate SOA, that is at SOA 100, 41 ms versus 27 ms, *t*(22) = 2.694, *p* < .013, SOA 300, 51 ms versus 38 ms, *t*(22) = 3.049, *p* < .006, and SOA 900, 18 ms versus − 2 ms, *t*(22) = 2.784, *p* < .011, respectively. The larger amount of the reward-related reduction of RT1 compared to RT2 is consistent with the assumption that reward affected at least partially the motor processes of task 1 in addition to its effects on pre-and/or bottleneck stages in task 1; while the latter effects cause a shortening in the task 1 processing chain, which propagates via the bottleneck from task 1 to task 2, the reward leads to an additional shortening of the RT1, which is not reflected in corresponding RT2 effects.

We also found a significant interaction of the factors Reward × SOA, *F*(2, 44) = 4.561, *p* <. 016, η_p_² = 0.172, demonstrating larger reward effects at SOA 100 (*m =* 34 ms) compared to SOA 900 (*m* = 8 ms), *t*(22) = 2.048, *p* < .026. In addition, we found a significant interaction of the factors Task × SOA, *F*(1.037, 22.816) = 295.243, *p* < .001, η_p_² = 0.931. Naturally, task 2 was stronger affected by the SOA manipulation, than task 1. The interaction of the factors Task × Reward × SOA did not reach significance, *F*(1.580, 34.763) = 0.542, *p* = .545, η_p_² *=* 0.024.

**Table 2:** Mean reward effects for task 1 and task 2 in ms (and standard deviation) from experiment 1 as a function of SOA

			Experiment 1				
		Reward effects					
		Task 1			Task 2		
SOA							
100		41 ms (11 ms)			27 ms (10 ms)		
300		51 ms (11 ms)			38 ms (11 ms)		
900		18 ms (10 ms)			-2 ms (6 ms)		

### Discussion

In Experiment 1, the direct reward application to participants’ task 1 performance, reduced both RT1 and RT2, which is reflected by the main effects of reward on RT1 as well as on RT2. The observation of an overadditive interaction of SOA and reward on RT2 reflects larger reward effects on RT2 at short compared to long SOA, which is consistent with the assumption that (at least part of) the reward effect propagated at short SOA from task 1 onto task 2, thus reducing RT2. This effect pattern demonstrates that the pre-and/or bottleneck processing stages of task 1 were shortened by the direct reward application to task 1 and that the observed reward effects on task 2 are the result of effect propagation via the bottleneck between tasks. A further hint for effect propagation stems from the analysis of the interdependency of the response times in task 1 and task 2 (Pashler & O’Brien, 1993), which we will discuss in more detail together with related findings in Experiment 2.

Importantly, we furthermore obtained results in line with the assumption that the reward prospect for task 1 performance results in larger reward effects on task 1 compared to task 2. This pattern was observed for each SOA level, providing evidence that not the entire task 1 reward effect was propagated from task 1 to task 2. Based on the predictions of the effect propagation logic (Pashler & Johnston, 1989; Schubert, 1999; Schweickert, 1978; Van Selst et al., 1999; Van Selst & Jolicoeur, 1997) these findings are consistent with the assumption that the prospect of reward for task 1 performance also affected the motor processes of task 1.

In the next experiment, we aimed to assess over which processing stages of task 2 the reward-related processing time reduction of pre-and/or bottleneck stages in task 1 will be propagated onto the task 2 processing chain. In other words, we asked which processing stages of task 2 are processed earlier due to the reward prospect onto task 1 processing.

## Experiment 2

The aim of Experiment 2 was to identify the task 2 processing stage, which is processed earlier due to the reward prospect on task 1. For that purpose, we localized the bottleneck in the processing chain of a PRP task, while additionally, applying a reward prospect to task 1 processing. For the bottleneck localization, we applied a difficulty manipulation of the response selection stage of task 2, resulting in easy (compatible response mapping) and hard (incompatible response mapping) conditions (McCann & Johnston, 1992). Combining the locus-of-slack and effect propagation logics enabled us to distinguish whether the reward effect propagated over the central or peripheral bottleneck from task 1 to task 2 (Johnston & McCann, 2006; McCann & Johnston, 1992; Pashler & Johnston, 1989; Schubert, 1999; Schubert et al., 2008). For that matter, we will outline the predictions of the response selection difficulty manipulation and SOA on RT2 in particular, while the effects of reward and SOA will be discussed separately.

**Fig. 4:**
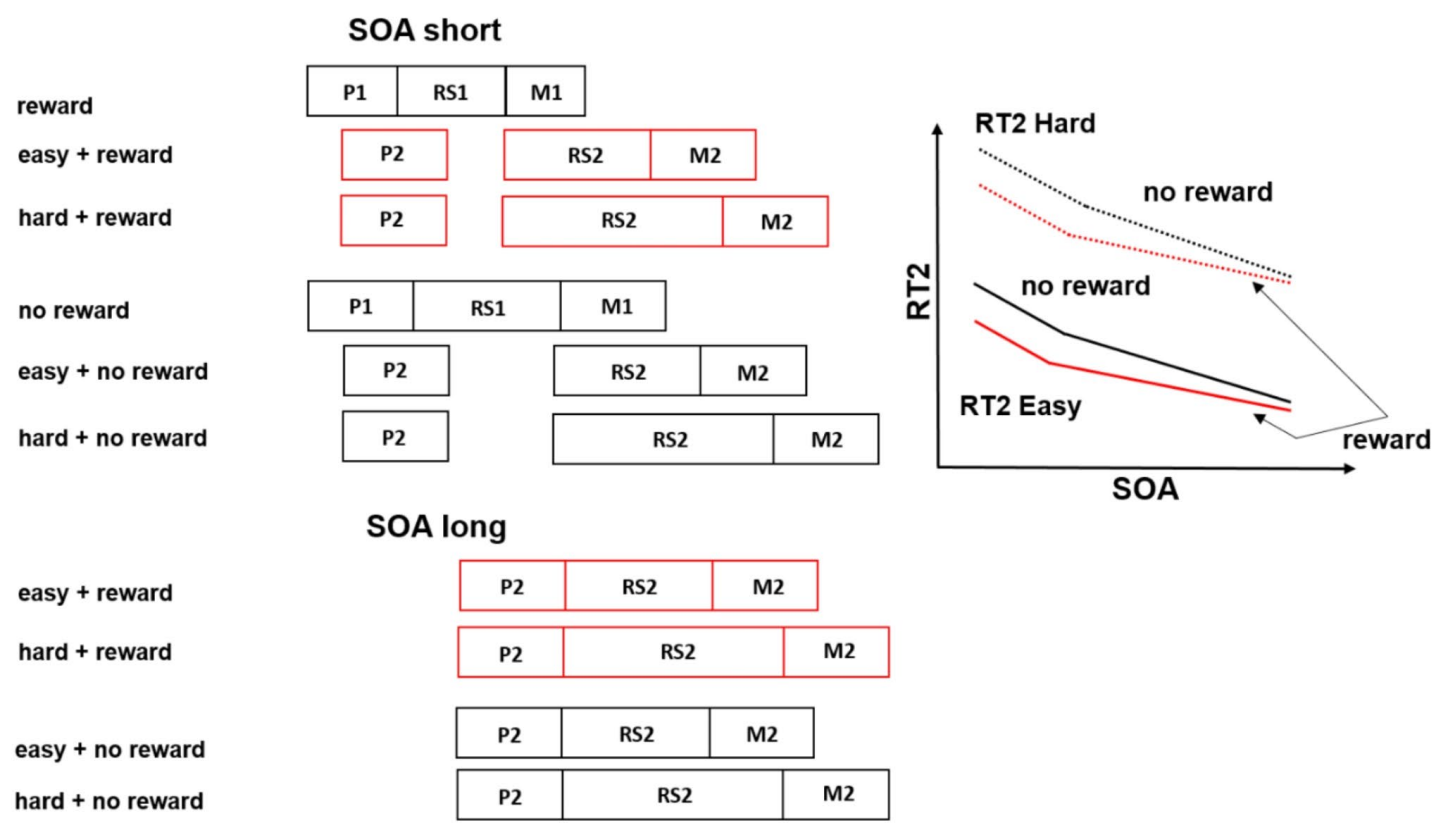
Response selection bottleneck model including reward influencing the pre-and/or bottleneck and the post-bottleneck stages of task 1. Furthermore, the difficulty manipulation of the response selection of task 2 and RT2 predictions are depicted: Additive effects of the difficulty manipulation and SOA on RT2 should emerge, if the response selection stages of both tasks are processed serially, favoring the response selection bottleneck model (Easy = rule-based stimulus-response mapping; Hard = arbitrary stimulus-response mapping; Red indicates the rewarded conditions at short and long SOA respectively; P1 = perception stage of task 1; RS1 = response selection stage of task 1; M1 = Motor stage of task 1; P2 = perception stage of task 2; RS2 = response selection stage of task 2; M2 = motor stage of task 2)

First of all, let us consider how the RT2 pattern should look like if a bottleneck at the response selection stage would interrupt the processing chain of task 2. In that case, the RT2 in the hard condition should be increased compared to the easy condition. This is observable in Fig. 4, which indicates additive effects of the response selection difficulty manipulation and SOA on RT2. In particular, in the hard condition, RT2 should be increased during short and long SOA by the *same* amount of additional time, since the additional time is added *after* the response selection bottleneck. Concerning the reward effects, we expect to replicate the findings from Experiment 1. That is, we expect to find that reward affects the processing stages before or at the bottleneck of task 1, leading to effect propagation on task 2 at short SOA. This can be seen in Fig. 4, which illustrates an overadditive interaction of SOA and reward on RT2. Such a reward effect pattern would be accompanied by the additive effects of SOA and response selection difficulty on RT2.

**Fig. 5:**
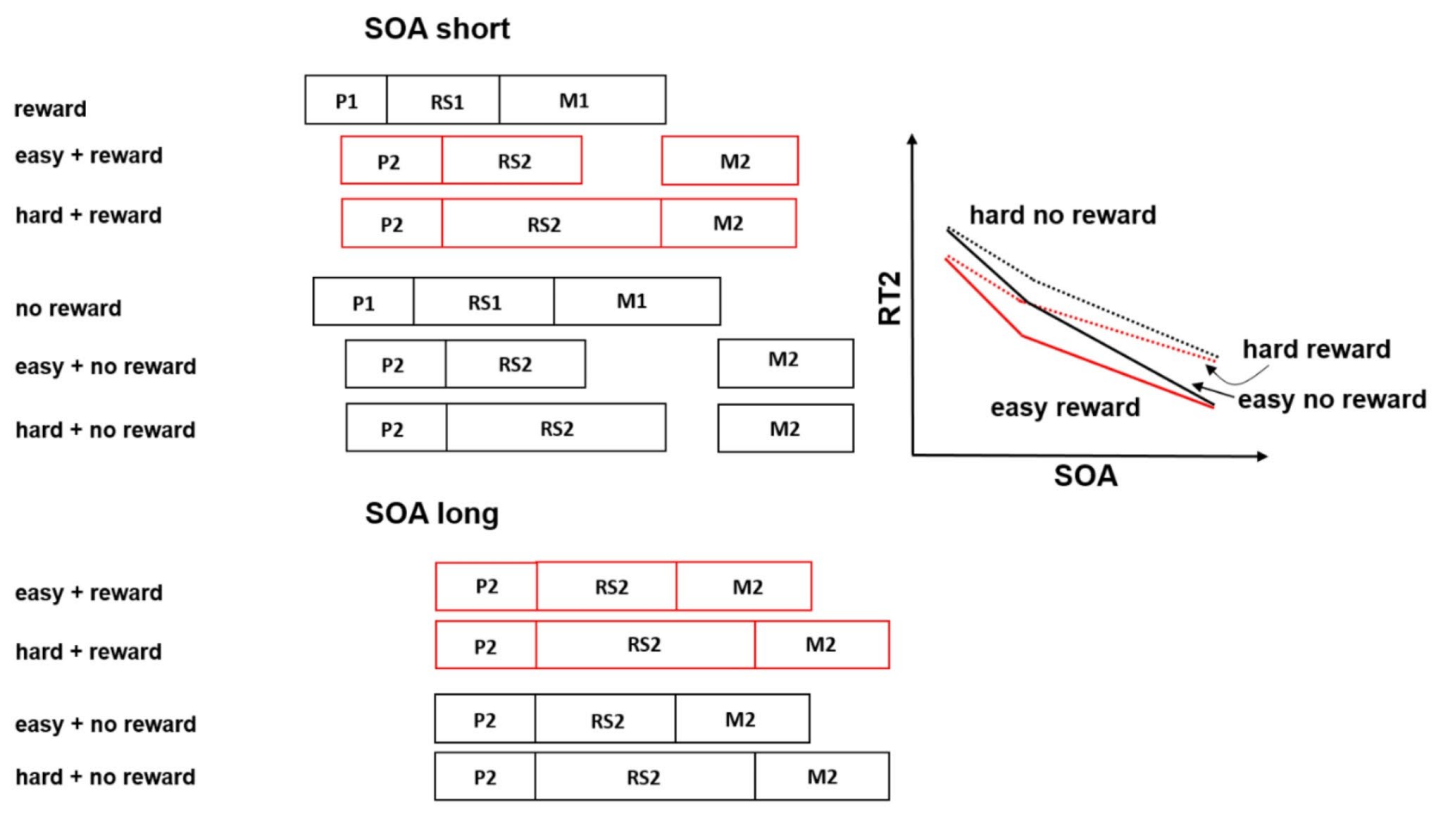
Response initiation bottleneck model including reward influencing the pre-and/or bottleneck and the post-bottleneck stages of task 1. Furthermore, the difficulty manipulation of the response selection of task 2 and RT2 predictions are depicted: Underadditive effects of the difficulty manipulation and SOA on RT2 should emerge if the response selection stages of both tasks are processed concurrently, favoring the response initiation bottleneck model (Easy = rule-based stimulus-response mapping; Hard = arbitrary stimulus-response mapping; Red indicates the rewarded conditions at short and long SOA respectively; P1 = perception stage of task 1; RS1 = response selection stage of task 1; M1 = Motor stage of task 1; P2 = perception stage of task 2; RS2 = response selection stage of task 2; M2 = motor stage of task 2)

However, how should the RT2 pattern look like if a bottleneck would interrupt the processing chain at the response initiation stages between task 1 and task 2? If that were the case, we would expect an underadditive interaction of the response selection difficulty manipulation and SOA on RT2. As can be seen in Fig. 5, the additional time needed in the hard compared to the easy condition would be absorbed into the slack at short SOA, but not during long SOA. In particular, during long SOA, we would expect an increased RT2 for the hard compared to the easy condition, however, no differences between both conditions during short SOA on RT2. Regarding reward processing, we predict a replication of the results from Experiment 1. That is, reward should affect the processing stages before or/at the bottleneck of task 1, which would lead to effect propagation at short SOA on task 2, but not at long SOA. As can be seen in Fig. 5, we expect an underadditive interaction of response selection difficulty and SOA, as well as, an overadditive interaction of reward and SOA on RT2.

Important to note, that for both hypotheses about bottleneck location mentioned before we would expect reward effects on the motor stage of task 1, which are not propagated to task 2 (i.e., increased reward effects on task 1 compared to task 2).

### Materials and methods

#### Participants

Twenty-four healthy participants (20 female; *mean* (*m*) *age = 20.5* years) were invited to take part in the experiment after obtaining written informed consent. Participants could choose between 4 Euro or course credit as base payment. The experimental protocol conformed to the declaration of Helsinki. All participants were right-handed, German native speakers, and had normal or corrected to normal vision.

#### Apparatus and stimuli

The apparatus and the stimuli were the same as in Experiment 1. For the easy condition, we employed the same stimulus-response mapping as in Experiment 1. For the hard condition, we used an arbitrary (rather than a compatible) stimulus-response mapping for the visual task (task 2). In this condition, participants responded to the digits 1, 5, and 9 by pressing the ‘.’, ‘-, and ‘,’ buttons of a QWERTZ keyboard with the middle, ring, and index finger of their right hand. The difficulty manipulation was applied per block, resulting in easy and hard blocks, respectively. The trial sequence was identical to Experiment 1 with the exception that we included an additional SOA of 50 ms and adjusted the SOA of 100 ms to 150 ms. We aimed to investigate in more detail the time course of RT2 over the temporal overlap of both tasks. Participants received the feedback “Falsch” (German for wrong) for 500 ms if either one or two of their responses were erroneous. If their response to either target exceeded the maximal response duration, the feedback “Zu langsam” (German for too slow) was presented for 500 ms. For Experiment 2, the computations for the reward threshold in the easy and hard conditions were identical to the computations applied in Experiment 1.

#### Design and procedure

A three-factor within-subjects design with SOA and reward and compatibility as independent variables were used. Each block consisted of 36 trials resulting from the combination of 4 SOAs (50, 150, 300, 900 ms), 3 auditory stimuli (250, 500, 1000 Hz), and 3 visual stimuli (1, 5, 9). Reward was varied blockwise. In total, there were 16 blocks: 4 blocks of reward/easy mapping, and 4 blocks of reward/hard mapping. As well as, 4 blocks of non-reward/easy mapping, and 4 blocks of non-reward/hard mapping which resulted in an overall of 576 trials. The procedure was analogous to Experiment 1. After 24 single-task practice trials (12 for each component task) participants performed two runs of 36 trials of DT practice. The first run was the easy DT block and the second run was the hard DT block. The reward instruction, as well as the computation for the reward thresholds for obtaining a reward, were identical to Experiment 1.

#### Statistical analysis

We analyzed mean RTs and error rates separately for RT1 and RT2 using an ANOVA with the within-subjects factors SOA, reward, and task 2 difficulty. A significance threshold of 5% was used for all analyses. The *p* values of the ANOVAs were adjusted according to the Greenhouse-Geisser correction when necessary. For the RT analyses, trials with at least one erroneous response (*m* = 10%) and outliers that deviated more than +/- 2.5 SD (*m =* 2% ) were excluded from the data set. Furthermore, trials were excluded that met the criterion of response grouping (RT2 – RT1 + SOA) < 200 (Miller & Ulrich, 2008).

### Results

#### Task 1

**Fig. 6:**
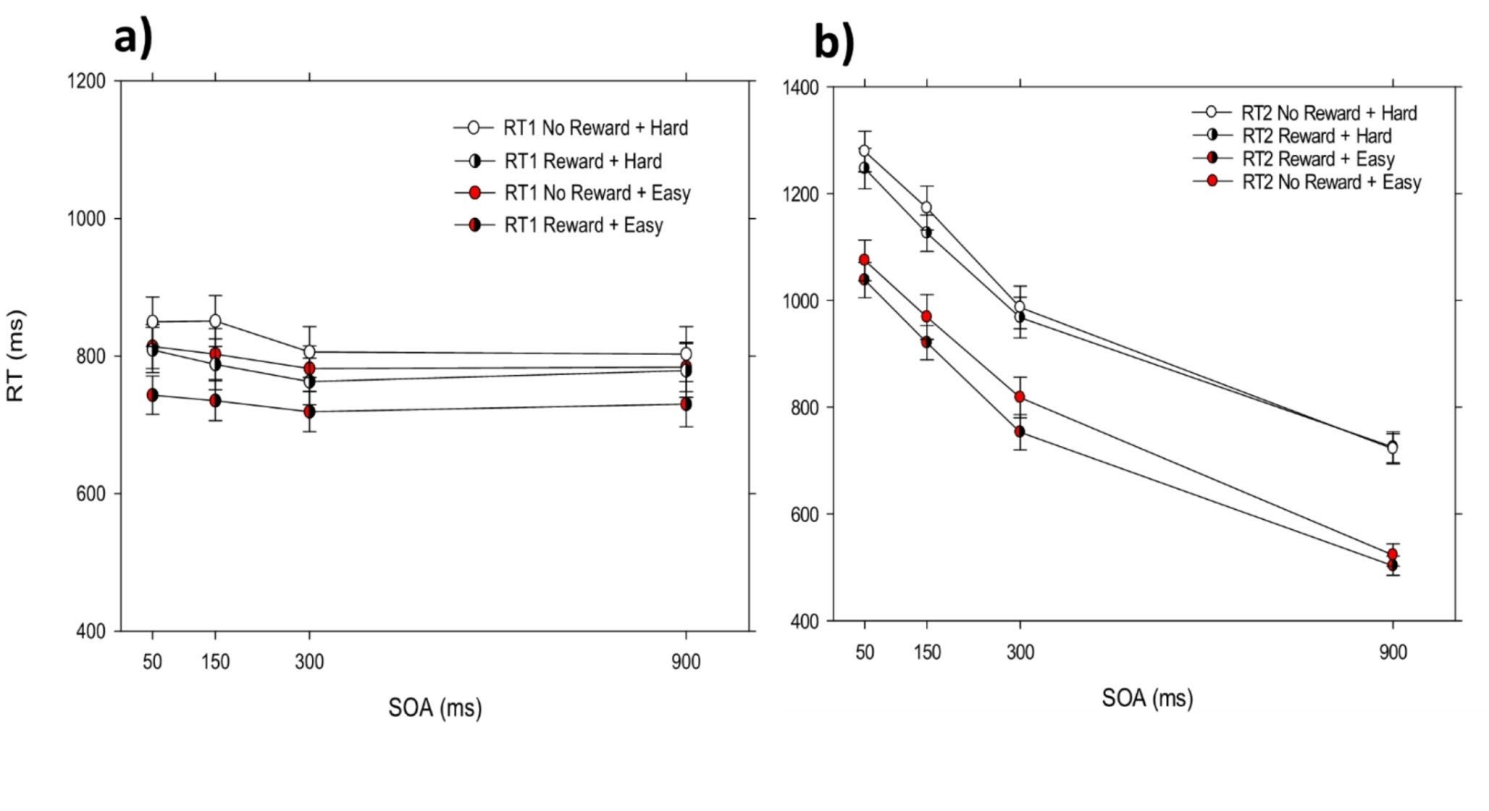
Mean RT1 and RT2 as a function of SOA, reward, and compatibility for Experiment 2. Panel (**a**) represents task 1 performance and panel (**b**) represents task 2 performance. Error bars represent the standard error of the mean

Similar to Experiment 1, in Experiment 2 we found a significant main effect of reward on RT1, *F*(1, 23) = 17.560, *p* < .001, η_p_² = 0.433. RT1 was shorter in the reward (*m* = 758 ms) compared with the no reward condition (*m* = 812 ms) (see Fig. 6a). Furthermore, we found a significant main effect of the factor compatibility on RT1, *F*(1, 23) = 22.756, *p* < .001, η_p_² = 0.497, indicating shorter response times in the easy (*m* = 764 ms) than in the hard condition (*m* = 806 ms). In addition, we found a significant main effect of SOA on RT1, *F*(1.819, 23) = 3.410, *p* < .047, η_p_² = 0.129, pointing to a slight grouping tendency of participants (Ulrich & Miller, 2008). In addition, we observed a significant interaction of the factors Reward × Compatibility, *F*(1, 23) = 4.714, *p* < .040, η_p_² =0.170. With a larger reward effect in the easy (*m* = 64 ms) compared to the hard (*m* = 43 ms) condition, *t*(23) = 2.171, *p* <. 040.

Neither the interaction of the factors Reward × SOA, *F*(3, 69) = 1.942, *p* = .131, η_p_² = 0.078, nor the interaction of the factors Compatibility × SOA, *F*(3, 69) = 1.155, *p* = .333, η_p_² = 0.048, nor the three-way interaction of the factors Reward × Compatibility × SOA, *F*(3, 69) = 0.321, *p* = .810, η_p_² = 0.014, reached significance.

For the error rates in task 1, we observed a significant effect of the factor compatibility, *F*(1, 23) = 16.307, *p* < .001, η_p_² = 0.451, which was modulated by the SOA between tasks, SOA × Compatibility *F*(3, 69) = 8.396, *p* < .001, η_p_² = 0.267. This reflects smaller compatibility effects (*m* = -4%) at SOA 50 compared to SOA 900 (*m* = 0.5%). Thus the error rates converged at SOA 900 in the hard and easy conditions.

Furthermore, we observed a significant effect of the factor reward, *F*(1, 23) = 5.336, *p* < .030, η_p_² = 0.188. Participants committed fewer errors in the reward (*m* = 7%) compared to the no reward condition (*m* = 8%). In addition, we obtained a significant effect of the factor SOA, *F*(1.894, 69) = 17.462, *p* < .001, η_p_² = 0.432. The error rates increased from SOA 900 (*m* = 5%) to SOA 50 (*m* = 10%).

Neither the interaction of the factors Reward × Compatibility, *F*(3, 69) = 1.054, *p* = .315, η_p_² = 0.044, nor the interaction of the factors, Reward × SOA, *F*(3, 69) = 1.404, *p* = .249, η_p_*²* = 0.058, nor the threeway interaction of the factors Reward × Compatibility × SOA, *F*(3, 69) = 0.353,, *p* = .787, η_p_² = 0.015, reached significance.

#### Task 2

We found a significant main effect of the factor SOA, *F*(3, 23) = 590.683, *p* < .001, η_p_² = 0.963. RTs increased from SOA 900 (*m* = 618 ms) to SOA 50 (*m* = 1160) ms indicating a typical PRP effect. Similarly to Experiment 1, we found a significant main effect of the factor reward on RT2, *F*(1, 23) = 8.250, *p* < .009, η_p_² =0.264 (see Fig. 6b). RT2 was reduced in the reward condition (*m* = 910 ms) compared with the no reward condition (*m* = 943 ms). This main effect was further specified by the Reward × SOA interaction, *F*(2.149, 69) = 3.188, *p* < .046, η_p_² = 0.122. Pairwise comparisons revealed an overadditive interaction of SOA and reward on RT2 with a larger reward effect on task 2 at SOA 50 (*m* = 34 ms ) compared with SOA 900 (*m* = 8 ms), *t*(23) = 2.515, *p* < .019. Furthermore, the reward effect was not different for SOA 150 and SOA 300, t(23) = 0.352, *p* = .362; similarly, the reward effect did not differ between SOA 50 and SOA 300, t(23) = − 0.664, *p* = 257.

Furthermore, we observed a significant main effect of the factor compatibility, *F*(1, 23) = 168.084, *p* < .001, η_p_² = 0.880. RT2 was shorter in the easy condition (*m* = 825 ms) compared with the hard condition (*m* = 1028 ms). Most important for the issue of the bottleneck location, we found no significant Compatibility × SOA interaction, *F*(3, 69) = 2.686, *p* = .115, η_p_² = 0.105, which is consistent with the assumption that the SOA and the compatibility manipulation affected the RT2 in an additive manner and speaks against the assumption of a bottleneck at the response initiation stage in the current study. Instead, it points to serial scheduling of the response selection stages and to a response selection bottleneck.

Neither, the interaction of the factors Reward × Compatibility, *F*(1, 23) = 2.686, *p* = .115, η_p_² = 0.105, nor the three-way interaction of Reward × Compatibility × SOA, *F*(3, 69) = 0.831, *p* = .481, η_p_² = 0.035, reached significance.

For the error rates in task 2, we found a significant main effect for the factor compatibility, *F*(1, 23) = 57.209, *p* < .001, η_p_² = 0.713. Participants committed more errors in the hard (*m* = 13%) compared to the easy (*m* = 8%) condition. We, furthermore, obtained a significant effect of the factor reward, *F*(1, 23) = 4.392, *p* < .047, η_p_² = 0.160. The error rates were decreased in the reward (*m* = 8%) compared to the no reward (*m* = 10%) condition. In addition, we observed a significant interaction of the factors Reward × Compatibility, *F*(1, 23) = 13.190, *p* < .001, η_p_² = 0.364. The compatibility effect was increased in the no reward (*m* = 9%) compared to the reward (*m* = 5%) condition, *t*(23) = -3.632, *p* <. 001. We also observed a significant interaction of the factors SOA × Compatibility, *F*(2.221, 69) = 5.566, *p* < .005, η_p_² = 0.195. Pairwise comparisons revealed that this interaction was mostly driven by significant differences between error rates across different SOAs in the easy response selection condition, while the error rates hardly differed between SOAs in the hard response selection condition. In detail, the error rate in the easy condition at long SOA 900 (*m* = 4%) was significantly reduced compared to that at SOA 50 (*m* = 6%), and at SOA 300 (*m* = 6%), both *ts*(23) > -2.74, *ps* < 0.001, while it was numerically but not significantly smaller than that at SOA 150, *p* > .05. In contrast, the error rate in the hard condition at SOA 900 (*m* = 15%) was not significantly different from that at SOA 50 (*m* = 12%), *t*(23) = -1.7, *p* > .09, and SOA 300 (*m* = 12%), *t*(23) = 0.824, *p* > .40, while the difference approached significance compared to that at SOA 150, (*m* = 11%), *t*(23) = 2.02, *p* = .054.

In sum, this pattern results in a smaller compatibility effect (hard minus easy) of error rates at short SOA (*m* = 6%) compared to that at long SOA (*m* = 11%), *t*(23) = 2.939, *p* < .01, which however, was especially caused by the significant reduction of error rates in task 2 at SOA 900 compared to all other SOAs in the easy response selection condition but not in the hard condition.

The factor SOA did not significantly affect the error rate, *F*(3, 23) = 0.582, *p* = .629, η_p_² = 0.025 (Mattes et al., 2021; Strobach et al., 2015a). Neither the interaction of the factors Reward × SOA, *F*(3, 69) = 1.811, *p* = .153, η_p_² = 0.073, reached significance, nor the three-way interaction of the factors Reward × Compatibility × SOA, *F*(3, 69) = 0.310, *p* = .818, η_p_² = 0.013. The error rates for Experiment 2 can be found in Table 3.

**Table 3:** Mean rates of errors for task 1 and task 2 in % (and standard deviation) from experiment 2 as a function of reward, SOA, and compatibility

			Experiment 2						
	Compatibility - Reward								
	easy - reward		easy – no reward		hard - reward			hard - no reward	
SOA	Task 1	Task 2	Task 1	Task 2	Task 1	Task 2		Task 1	Task 2
50	11.00% (9.21%)	6.71% (4.77%)	14.24% (7.61%)	5.79% (4.01%)	6.94% (6.60%)	11.22% (7.92%)		9.49% (6.99%)	13.19% (6.87%)
150	8.80% (7.49%)	6.13% (5.67%)	10.19% (5.83%)	5.43% (4.81%)	5.67% (6.37%)	9.84% (9.41%)		5.55% (4.85%)	12.96% (8.14%)
300	6.37% (5.01%)	6.02% (5.66%)	7.52% (5.46%)	6.83% (5.05%)	4.16% (4.18%)	10.88% (9.58%)		5.79% (4.70%)	14.00% (8.69%)
900	4.28% (5.24%)	3.82% (4.32%)	5.56% (4.56%)	4.62% (4.54%)	5.56% (4.10%)		12.27% (9.65%)	5.44% (4.81%)	17.59% (9.85%)

### Relationship between RT1 and RT2

The subsequent analysis focused on the relationship between RT1 and RT2 to investigate effect propagation from task 1 onto task 2 in more detail. As for Experiment 1, we predicted an interaction of SOA and quintile on RT2 indicating effect propagation from task 1 onto RT2 at short but not at long SOA. For this investigation, we relied on an approach established by Pashler and O’Brien (1993).

Figure 3b depicts that an increase in RT1 leads to a rise in RT2 as well. Most importantly, as SOA was reduced from 900 ms to 50 ms, the dependency of RT2 on RT1 increased. This observation was confirmed by the results of an ANOVA with RT2 as the dependent variable and the factors reward, SOA, and RT1 broken into quintiles (collapsed over two compatibility conditions for reasons of simplicity). Here, we obtained a significant interaction of the factors SOA × Quintile, *F*(12, 276) = 44.63, *p* < .001, η^2^ = 0.088.

Moreover, as depicted in 3b and following the results from Experiment 1 we obtained a significant interaction of the factors Reward × SOA, *F*(3, 69) = 3.08, *p* < .033, η^2^ = 0.002. Such a result suggests larger reward effects at shorter compared to longer SOAs on RT2 and replicates the findings obtained from our previous analysis of task 2 performance across both experiments.

Furthermore, we found several further effects. Figure 3b shows increased reward effects for larger RTs compared to smaller RTs, this observation was confirmed by an interaction of the factors, Reward × Quintile, *F*(4, 92) = 5.78, *p* < .001, η^2^ = 0.004. As in Experiment 1, such an effect could indicate that reward reduces lapses of attention, which usually occur during longer RTs.

In addition, we obtained a significant main effect of the factor SOA, *F*(3, 69) = 554.96, *p* < .001, η^2^ = 0.582 and a main effect of the factor quintile, *F*(4, 92) = 152.88, *p* < .001, η^2^ = 0.390. The factor reward reached significance, *F*(1, 23) = 7.85, *p* < .010, η^2^ = 0.007, reflecting shorter RTs in the reward compared to the no-reward condition.

### Comparison of reward effects on task 1 and task 2 performance

We, again as in Experiment 1, tested whether the reward prospect on task 1 affected the post-bottleneck stage of task (1) For that matter, we compared the reward-related RT reductions on task 1 and task (2) To this end, we calculated a separate ANOVA across the RTs with the factors task (task 1 vs. task 2), SOA, and reward (collapsed together for the two compatibility conditions for reasons of simplicity). The factor task reached significance, *F*(1, 23) = 21.799, *p* < .001, η_p_² = 0.487, reflecting shorter RTs for task 1 (*m* = 785 ms) compared to task 2 (*m* = 927 ms). The factor reward reached significance, *F*(1, 23) = 14.032, *p* < .001, η_p_² = 0.379, showing shorter RTs in the reward (*m* = 834 ms) compared to the no-reward condition (*m* = 877 ms). In addition, we obtained a significant effect of the factor SOA, *F*(1, 23) = 247.948, *p* < .001, η_p_² = 0.915.

Most importantly, as in Experiment 1, we obtained a significant interaction of the factors Reward × Task, *F*(1, 23) = 7.331, *p* < .013, η_p_² = 0.379, which expresses the observation of larger reward effects on RTs in task 1 (*m* = 53 ms) compared to task 2 (*m* = 33 ms), *t*(23) = 2.708, *p* < .013. An additional, analysis of the separate SOAs further showed that reward led to a larger reduction of RT1 compared to RT2, during each SOA, except at SOA 300. For SOA 50, 56 ms versus 34 ms, *t*(23) = 2.708, *p* < .032, SOA 150, 66 ms versus 47 ms, *t*(23) = 2.730, *p* < .012, SOA 300, 53 ms versus 42 ms, *t*(23) = 1.097, *p* = .284, SOA 900, 39 ms versus 8 ms, *t*(23) = 2.621, *p* <. 015, respectively. The increased reward-related reduction of RT1 compared to RT2 is in line with the assumption that the prospect of reward affected the motor processes of task 1 in addition to its effects on the pre-and/or bottleneck processing stages of task 1. While the former reward effect is not carried over from the processing chain of task 1 to task 2, the latter reward effect propagates via the bottleneck from task 1 to task 2, thereby reducing RT2.

In addition, we obtained a significant interaction of the factors Reward × SOA, *F*(1, 23) = 2.926, *p* <. 040, η_p_² = 0.113. Pairwise comparisons indicated larger reward effects at SOA 50 (*m* = 34 ms) compared to SOA 900 (*m* = 8 ms), *t*(23) = 2.515, *p* <. 019. Furthermore, we obtained a significant interaction of the factors Task × SOA, *F*(1.258, 28.940) = 454.893, *p* <. 001, η_p_² = 0.952. This indicates that task 2 was strongly affected by the SOA application. The threeway interaction of Reward × Task × SOA did not reach significance, *F*(3, 69) = 1.324, *p* = .273, η_p_² = 0.054.

**Table 4:** Mean reward effects for task 1 and task 2 in ms (and standard deviation) from experiment 2 as a function of SOA and compatibility

			Experiment 2				
		Reward effects					
		Task 1			Task 2		
SOA							
50		56 ms (14 ms)			34 ms (15 ms)		
150		66 ms (16 ms)			47 ms (16ms)		
300		53 ms (15 ms)			42 ms (14 ms)		
900		39 ms (13 ms)			8 ms (11 ms)		

### Discussion

In Experiment 2, the reward application to task 1 led to reduced RT1 and RT2. Similarly, as in Experiment 1, we obtained an overadditive interaction of reward and SOA on RT2, which was accompanied by larger reward effects at short compared to long SOA on RT2. This pattern is consistent with the assumption that (at least part of) the reward effect propagated at short SOA from task 1 onto task 2, leading to a subsequent shortening of the RT2. This effect pattern indicates that the reward prospect to task 1 leads to a shortening of pre- and/or bottleneck-processing stages of task 1 and that the obtained reward effects on task 2 are the result of effect propagation over the bottleneck between tasks. As in Experiment 1, we obtained a robust interdependency of the response speed to task 2 on the response speed to task 1 at short SOA which was reflected by the interaction of quintile and SOA on RT2. In accordance with Pashler and O’Brien (1993), the interdependency of RT2 on RT1 was reduced with increasing SOA between tasks, as no bottleneck emerges in a PRP task with long SOA. Similar to Experiment 1, the results provide strong evidence for the assumption that effect propagation between task 1 and task 2 has taken part (at least at short SOAs), thus explaining how reward prospect on task 1 could lead to a reward-related reduction of task 2 processing time.

As a main question of Experiment 2, we investigated over which task 2 processing stages the reward-related processing time reduction in task 1 propagates into the processing chain of task 2. To tackle this question, we applied a difficulty manipulation of the response selection stage in task 2 and localized the bottleneck between tasks (McCann & Johnston, 1992; Pashler & Johnston, 1989; Schubert, 1999). The obtained pattern of results showed additive effects of the difficulty manipulation and of the SOA on RT2, which is consistent with the assumption that the bottleneck occurred at the response selection (McCann & Johnston, 1992; Schubert, 1999) and not at the motor response stage (Keele, 1972; Kieras & Meyer, 1997; Mittelstädt et al., 2022). This, in turn, indicates that the reward-related processing time reduction in task 1 propagated from the pre- and/or bottleneck stages of task 1 via a response selection bottleneck onto the processing chain of task 2, thus leading to an earlier onset of the response selection stage of task 2.

Please note that we obtained a significant interaction of the factors SOA x compatibility on the error rates of task 2, which might be interpreted as compromising the conclusion of a response selection bottleneck emerging in Experiment 2^2^. However, in our view, the observed interaction does not compromise the interpretation of the additive RT2 effects of SOA and compatibility as evidence for serial response selection processing in the two tasks. If the significant SOA x compatibility error rate interaction had been caused by parallel response selection processes at short SOA, then one should have expected improved task 2 processing, i.e. a decreased error rate, at short SOA compared to long SOA especially for the hard condition. This is so because the additional processing demands for the hard response selection should have been absorbed into slack, thus, causing more success when selecting the required response alternative compared to the situation at long SOA where no absorption of additional response selection demands is possible; please note, if the RTs have not benefited in the hard response selection condition at short SOA, then the improvement should have been expressed in the error rates. However, the error rate in the hard condition at short SOA 50 was not different from that at SOA 900 and it was larger in the hard compared to the easy response selection (SOA 50), which opposes the idea that any additional response selection processes had been absorbed into slack at short SOA. Instead, the particular error rate pattern across different SOAs suggests that the SOA x compatibility interaction was driven by a decreased error rate especially in the easy response selection condition at long SOA compared to all other SOAs. Various reasons could be proposed for explaining this pattern. For example, it would be consistent with the assumption that participants could more successfully prepare for task 2 in the easy response selection condition at long SOA compared to the other SOAs, where the two task chains are temporally overlapping to a larger degree. Since at the same time, the error rates in the hard response selection condition did not differ across SOAs, we believe that this is a more plausible explanation for the observed SOA x compatibility error rate interaction than the assumption of parallel response selection processes at short SOA (see also Schubert, 1999).

As a further important finding, we observed larger reward effects on RT1 compared to RT2 at each SOA level (except for SOA 300). This suggests that reward effects were located on pre-bottleneck and/or bottleneck stages and, in addition, at the motor stage of task 1. This is so because according to the effect-propagation logic any change of RT1 processing time, which occurs *after* the bottleneck in task 1, i.e. at post-bottleneck stages, would not result in corresponding RT2 changes. The observed pattern of a larger reward-related reduction of RT1 compared to RT2 is consistent with the assumption that part of the reward-related task 1 reduction occurred at post-bottleneck stages. Probably, the reward prospect on task 1 leads to improved execution of the task 1 motor response, which is expressed by shorter motor execution occurring after the bottleneck and leading to a shortening of the RT1 which is not expressed in a corresponding RT2 shortening.

## General discussion

The present study investigated the effects of a direct reward application to task 1, as well as, the question of which processing stages in the DT processing chain are affected by the prospect of reward. For this purpose, in Experiment 1, we applied a reward manipulation to participants’ task 1 performance in a PRP task situation. The results showed shorter RT1 in the reward compared to the non-reward condition across all SOA conditions. In addition, we also observed an overadditive interaction of SOA and reward onto RT2. According to the effect propagation logic, these results are consistent with the assumption that the reward prospect onto task 1 leads to a shortening of task 1 processes and that (at least part of) the processing time shortening is transmissed via the bottleneck onto task 2 processing time, and, thus, spills over to the non-rewarded task 2. In addition, reward on task 1 led to significantly larger reward effects on task 1 compared to task 2, which is consistent with the assumption that part of the reward effect affected those processes of task 1, which are located after the bottleneck and the reduction of which cannot be propagated to the task 2 chain.

In Experiment 2, we specified the processes of task 2, over which the reward-related task 1 processing advantage is transmissed onto the task 2 processing chain. As a result, the application of the locus-of-slack technique provided findings consistent with the assumption that the reward-related reduction of task 1 processing time is transmissed via the response selection processes from task 1 to task 2.

### The localisation of reward-related improvement in Task 1 processing in dual tasks

The current results are consistent with the assumption that the prospect of reward on task 1 affected both, the processes before and/or at the bottleneck and the post-bottleneck processes in task 1, i.e. the motor stages. The reward-effect localization at these processing stages in a DT situation extends the findings of other studies, which showed reward-related improvements of these processes but in single-task situations. For example, several studies (Asutay & Västfjäll, 2016; Engelmann, 2009; Hübner & Schlösser, 2010; Kiss et al., 2009) indicated that the prospect of reward can improve early attentional and/or perceptual processes in choice RT single tasks. For the specific case of auditory perceptual processing (as the current task 1), Asutay and Västfjäll (2016) showed that reward-dependent attentional learning can affect the attentional selection and consequently the perceptual acuity in an auditory detection task. In particular, the authors asked participants to discriminate target tones from control tones while associating different reward probabilities with the control tones in a reward-learning phase of the experiment. The results showed that the perceptual sensitivity concerning tone discrimination changed tremendously depending on the reward probabilities during the learning period. The authors concluded, that the motivational value biased the auditory attentional selection of the auditory stimuli during task processing.

Thus, it is conceivable that in the current auditory task 1 situation, the prospect of reward resulted in increased attentional effort leading to enhanced quality of the auditory sensory processing. Such an effect localization is also supported by the current observation that the prospect of reward improved the accuracy of task 1 performance, which might reflect an increased rate of evidence accumulation in the reward condition, improving accuracy, as well as, RTs, in contrast to the non-reward condition. The resulting shortening in task 1 processing time would be propagated via the bottleneck to the processing time of task 2 and lead to its subsequent shortening. Important to note that an additional reward-effect localization at the task 1 response selection processes would also explain the observed propagation of the reward effect from task 1 to task 2 processes. An improvement of the response selection would be consistent with the results of several studies (Etzel et al., 2016; Kennerley & Wallis, 2009), which have shown that reward prospect may influence the updating and maintenance of task-relevant information in working memory, thus reducing the time for the response selection stage. In sum, the current findings are consistent with a localisation of a considerable portion of the reward effects on the joint processing time for perception and response selection in task 1, which explains the reward effect propagation to the task 2 processing time at short SOA.

Additionally, part of the task 1 reward effects are localized outside the pre-bottleneck and bottleneck processing time, which, to the best of our knowledge, is a new observation for the case of DT situations (see Langsdorf et al., 2022). In the current study, the task 1 reward prospect reduced the RT1 to a larger extent than RT2, which indicates that not all processing time reduction in the task 1 chain was transmissed to the task 2 chain. Since the results of Experiment 2 indicated that the bottleneck between tasks was located at the response selection, we locate the particular processing time that is not transmissed to the task 2 time, to the post-bottleneck processes, i.e. the motor processing of task 1.

The observation of a larger reward effect on task 1 compared to task 2 is in contrast to the effect pattern obtained by an earlier study of our group (Langsdorf et al., 2022). One possibility for the discrepancy between the findings of the current study and that of Langsdorf et al. (2022) is the different assignment of the reward prospect on task 1 in the current study and on task 2 in Langsdorf et al. (2022), which might have changed task processing and the resulting pattern of motivational influences between the two studies. While in the current study, the reward prospect was related to the task 1 processing chain, the reward prospect in Langsdorf et al. (2022) was related to the bottleneck-interrupted task 2 processing chain. Therefore, the reward prospect in the current study could have led to a direct influence on the task preparation even on motor processes, which was prevented in the Langsdorf et al. (2022) study because of the bottleneck.

The assumption that the reward prospect on task 1 might have caused a direct impact on motor stages only in case that the task processing is not interrupted by a bottleneck would be consistent with recent findings of neurophysiological investigations focussing on the neural activation during the performance of PRP tasks with neuroimaging methods (Stelzel et al., 2008; Wang et al., 2023). For example, Wang et al., 2023 showed increased functional connectivity between sensory areas and the default-mode network indicating that the neuronal processing of task 2 is suspended during task 1 processing in a PRP-like DT situation. In addition, Stelzel et al. (2008) could show that bottleneck processing in task 2 decreased the functional connectivity between sensory areas and later processing areas in task 2 at short compared to long SOA, which causes the RT2 to increase at shorter compared to long SOA. Thus it is conceivable that the improved reward-effect transmission to the motor stages of task 1 has occurred in the current study but not in the study of Langsdorf et al., 2022 because, in the latter study, the reward prospect was related to task 2, i.e. to the suspended task of the PRP situation.

### Reward effects in rewarded and non-rewarded tasks in multiple task situations

The current findings allow for a more elaborated discussion of the occurrence of reward-related spillover effects from rewarded to non-rewarded tasks in DT situations, which can also contribute to the understanding of the mixed evidence on reward-related spillover effects reported in other studies (e.g., Kleinsorge & Rinkenauer, 2012; Rieger et al., 2021; Umemoto & Holroyd, 2015).

In the previous study of Langsdorf et al. (2022) and the current investigation, the application of task-selective reward associations enabled us, to further elucidate spillover effects from the rewarded to the non-rewarded task in DT situations. In particular, we obtained increased reward effects for the rewarded task in contrast to the non-rewarded task across both studies, while also obtaining reward-related task improvements for the non-rewarded task. Importantly, the chronometric approach in combination with a PRP paradigm enabled the conclusion that the temporal overlap of the processing chains of both component tasks is crucial for the emergence of spillover effects, as indicated by the effect propagation between tasks at short SOA. In contrast, for the long SOA condition, no or less reward-related spillover effects occurred between both tasks. Therefore, the current study and the study of Langsdorf et al. (2022) provided conclusive evidence under which DT conditions reward-related spillover effects from the rewarded to the non-rewarded task will emerge.

The current findings extend previous results from a study by Rieger et al. (2021) who compared reward-induced preparation effects across DT paradigms. For the case of a PRP-like DT paradigm, the authors applied either a high reward to task 1 and a low reward to task 2, or vice versa. The authors reported that a high reward prospect to task 1 (compared to low reward prospect) led to reward effects on task 1 performance; whereas a high reward prospect to task 2 (compared to low reward prospect) did *not* result in reward effects on task 2 performance. The authors suggested that the *absence* of reward-related task 2 improvements in the PRP-like DT paradigm could be caused by the need to coordinate two motor responses, which might have impeded the reward-induced improvement of task 2 preparation.

An alternative reason for the absence of reward-related task 2 improvements might result from the consideration of the way how participants perceived a reward prospect on task 2 performance and the selection of trials for the analysis in Rieger et al. (2021). In particular, our findings demonstrated that the reward prospect to task 2 performance, leads to a shortening of the task 1 processing stages before or/at the bottleneck and this, leads subsequently, via effect propagation over the central bottleneck to a shortening of the task 2 processing time, thus, reducing RT2 (Langsdorf et al., 2022). However, Rieger et al. (2021) selected for their analysis of task 2 performance specifically those trials, in which participants did *not* respond to task 1 but only to task 2. This, however, causes that a potential reward effect can not be transmissed from task 1 via the central bottleneck mechanism onto the task 2 processing chain, as no first response was made; in other words, this might prevent the detection of a spillover of reward effects between tasks in the PRP DT task.

The current findings and those of Langsdorf et al. (2022) support an assumption according to which the preparation of two motor responses in the PRP task does NOT prevent the emergence but represents a decisive precondition for the emergence of a spillover of the reward effect on the non-rewarded task 2. The need to process two tasks in an overlapping manner with a bottleneck connecting the processing streams seems to represent a precondition for the transmission of reward-related task improvements between tasks in overlapping DT situations.

In that respect, the results of Kleinsorge and Rinkenauer (2012) need to be discussed who showed reward-related spillover effects to a non-rewarded task in a cued task-switching paradigm, in which the two tasks are processed sequentially but not in an overlapping manner. In particular, participants executed parity or magnitude judgments on digit stimuli, for which the performance in *one* of the tasks was rewarded, while the other task was *not* rewarded for the entire experiment, which resulted in a constant task-reward association for the whole experiment. Before digit onset, a task cue signaled to the participants which task to execute, while in some trials an additional cue signaled whether or not the current trial is a reward trial. Consequently, in some trials, the prospect of reward was signaled, but the possibility of receiving the reward was conditional upon whether the rewarded or the non-rewarded task should be executed. The authors reported improved task performance for the rewarded task if the cue signaled the prospect of reward compared to when no reward was signaled. However, task performance for the non-rewarded task was also improved, particularly, in those situations in which the cue signaled the prospect of a reward compared to no reward cues. Consequently, the cue signaling potential reward led to improved task performance for the rewarded task *and* the non-rewarded task as well. The authors suggested that the prospect of reward (as signaled by the cue) led to phasic alertness resulting in the mobilization of increased processing resources, which spilled over to improve task performance even in the non-rewarded task.

The conjoint discussion of the results on reward-related spillover effects in the PRP DT and in cued task-switching situations enables a further specification of the task conditions for which reward-related spillover effects are likely to emerge. The results of Kleinsorge and Rinkenauer (2012) suggest that the temporal coincidence of processes evoked by the reward cue *with* the task preparation to the non-rewarded task leads to the mobilization of increased processing resources that spilled over to improve performance in that (by definition) non-rewarded task. In fact, the results of the current study showed that the temporal overlap of the rewarded task 1 and the non-rewarded task 2 is important for enabling the transmission of reward effects between these tasks over the bottleneck, with increasing reward effects with short compared to long SOA, i.e. with larger compared to less temporal overlap. As a result, this indicates that a sufficient amount of temporal overlap of the reward prospect (i.e. either cued or task-related) with the processes in the preparation of the non-rewarded task represents an important precondition for the emergence of reward-related spillover effects in multiple task situations. Future studies should specify the temporal limitation for which an optimized reward-induced preparation can be achieved, i.e. by determining the optimal time range necessary for efficient spillover effects between rewarded and non-rewarded task processes.

Additionally, the emergence of reward-related improvements in DT situations is modulated by strategic influences resulting from the assignment of the specific reward association by the participants. In more detail, the comparison of the size of the reward effects in Langsdorf et al. (2022) and the current study indicates that depending on whether the prospect of reward was either associated with task 1 or task 2 performance, the magnitude of the reward effect was increased for the rewarded task compared to the non-rewarded task. Such an effect pattern indicates that participants do not handle the two component tasks as completely interrelated tasks but as two tasks with different and separate reward values, which leads to different outcomes of the reward effects in dual-task situations.

The differences in reward effects across both studies could be indicative of a strategic processing adjustment for the allocation of mental effort, to maximize rewarded task performance in order to receive a reward (Kool & Botvinick, 2018). In particular, if task 1 is rewarded but not task 2 (as is the case in the current study), participants allocate increased mental effort to the execution of task 1, which in turn results in larger reward effects on task 1 compared to task 2. On the contrary, if as was the case in Langsdorf et al. (2022) task 2 but not task 1 is associated with the prospect of reward, then participants should first also focus on the execution of task 1, because a fast task 1 execution would result in fast task 2 execution, as well, because of the bottleneck mechanism. The observation of a larger reward effect on task 2 indicates that participants maximized their allocation of mental effort by especially attending to task 2 processing.

From a broader perspective, the current findings, drawing on a careful application of chronometric inferences in overlapping DT situations, allow us to extend former conclusions about reward-cognition interactions in sensory-motor RT tasks. While earlier studies have often mainly focussed on issues like the reward-related modulation of e.g. conflict processing, attention, or cognitive flexibility (e.g., Fröber & Dreisbach, 2016; Jimura et al., 2010; Kiss et al., 2009; Krebs et al., 2010; Locke & Braver, 2008), the current results promote a different perspective; namely to analyze in detail and to compare the magnitude of the reward-related task improvements occurring across and between the separate tasks in multiple task situations. The application of analytic tools like the locus-of-slack technique (Pashler & Johnston, 1989; Schweickert, 1978) in combination with a careful manipulation of the reward prospects to different task streams might be fruitful for further pinpointing the question of task-specific reward effects and their transmission to the non-rewarded task chain.

### Reward effects and the question of parallel versus serial processing in dual tasks

An important further aspect is the question of serial versus parallel processing of the response selection processes in the two tasks and whether or not the application of monetary reward leads to a change in this architecture. Note that authors like Meyer and Kieras (1997) (see also Salvucci & Taatgen, 2008) propose that participants may engage in more daring dual-task coordination strategies leading to more parallel processing of the response selection processes under certain conditions, such as monetary reward. Other authors assume a central bottleneck causing serial scheduling of the response selection processes in the two tasks for structural reasons of a limited capacity for response selection processes (Pashler, 1994; Welford, 1959).

The current application of the response selection difficulty manipulation with the locus-of-slack technique in Experiment 2 allows us to test whether or not the application of reward has changed the serial scheduling of the response selection processes in the two tasks (McCann & Johnston, 1992). Importantly, the current results indicate that both response selection stages were processed serially constituting a central bottleneck and that the application of reward did not lead to more parallel processing of the central stages as could be assumed if considering the possibility of a strategic bottleneck processing in overlapping task processing (Meyer & Kieras, 1997; Salvucci & Taatgen, 2008). Instead, the current findings suggest that the central bottleneck processing has not changed due to reward prospects onto task 1 processing. This complements the findings of Langsdorf et al. (2022), who also showed that reward prospect onto task 2 processing does not change the bottleneck localization in a DT situation either. Thus, the combined consideration of the results of both studies suggests that monetary reward on either task 1 or task 2 improves DT processing, but does not lead to a change in the serial scheduling of response selection processing in overlapping dual tasks.

This is also in line with a study by Fischer et al. (2018) who reported improved serial processing in a PRP task situation due to reward prospect and used a different methodology in order to investigate parallel processing in a DT situation. In detail, the authors investigated the influence of reward prospect on the size of the backward compatibility effect (BCE), which reflects an influence of the congruence between the motor response in task 2 and task 1, with larger RT2 and RT1 in incongruent compared to congruent conditions. This can be explained by the occurrence of response activation processes for task 1 and task 2 motor responses, which operate simultaneously during the refractory period of both tasks (Hommel, 1998; Lien & Proctor, 2002; Schubert et al., 2008). Importantly, the authors observed a reduced BCE in the reward compared to the non-reward condition and interpreted this with the conclusion of a reduced amount of simultaneous response activation and an increased degree of serial DT processing. Thus, these findings just as the findings observed with the locus-of-slack technique in the current study do not support an assumption that reward prospect increases the amount of parallel processing of response selection in DT situations.

## Conclusion

We provided evidence that the prospect of reward for task 1 results in effect propagation over the central bottleneck from task 1 to the non-rewarded task 2, leading to an earlier onset of the response selection stage of task 2. Thus the effect propagation logic is applicable for the interpretation of the reward-related spillover effect between tasks. While the prospect of reward improved RT1 and RT2, the serial scheduling of the response selection stages was not altered. Importantly, parts of the reward effect were not propagated to task 2 thus affecting motor-related processes of task 1. As a result, the prospect of reward for task 1 performance led to increased reward effects on task 1 compared to task 2.

## Data Availability

The data generated and/or analyzed during the current study are available from the corresponding author upon reasonable request.

## Footnotes

1 Please, note that we calculated a similar power analysis for Experiment 2; however, with four SOA levels. This led also to an estimated sample size of *N* = 24.

2 This was proposed by an anonymous reviewer.
